# The intersection of melanoma survival and social determinants of health in the United States: A systematic review

**DOI:** 10.1016/j.jdin.2024.07.006

**Published:** 2024-08-02

**Authors:** McKenzie E. Maloney, Caleb Bacak, Kellen Tjioe, Loretta S. Davis, E. Andrew Balas, Gagan Agrawal, Jorge E. Cortes, Marisol Miranda-Galvis

**Affiliations:** a Medical College of Georgia, Augusta University, Augusta, Georgia; b Georgia Cancer Center, Augusta University, Augusta, Georgia; c Department of Dermatology, Medical College of Georgia, Augusta University, Augusta, Georgia; d Institute of Public and Preventive Health, Augusta University, Augusta, Georgia; e School of Computing, University of Georgia, Athens, Georgia

**Keywords:** disparities, economic stability, education, health care, melanoma, physical environment, skin cancer, social context, social determinants of health, survival

## Abstract

**Background:**

Despite recent improvements in melanoma survival rates, persistent inequalities pose barriers to care for some patients.

**Objective:**

To assess the influence of social determinants of health (SDoH) on melanoma treatment outcomes.

**Methods:**

A systematic review (Prospective Register of Systematic Reviews CRD42022346854) of manuscripts that examined the association between SDoH and melanoma treatment-related outcomes in the United States was conducted using 5 databases.

**Results:**

The analysis encompassed data from 12 retrospective manuscripts. The SDoH domains most frequently investigated were health care access and quality (*n* = 6 manuscripts, 50%) and economic stability (*n* = 7, 58.3%). Other domains included social and community context (*n* = 5, 41.7%) and education access (*n* = 3, 25%). These findings revealed significant correlations between poor melanoma survival and low levels of economic stability, limited education, government health insurance, and being uninsured and unmarried.

**Limitations:**

Many SDoH were not analyzed at the patient level. SDoH are vast categories, but manuscripts usually analyze one aspect of a particular category.

**Conclusions:**

These results highlight the need for physicians to recognize the substantial impact of SDoH on melanoma outcomes and to adopt more comprehensive strategies focused on patient-centered care. Integrating social support mechanisms into clinical practice emerges as a key mechanism to promote equitable and effective interventions.

**Capsule Summary**

• Social determinants of health, including low economic stability, government health insurance, absence of health insurance, limited educational attainment, and unmarried marital status, negatively impact melanoma survival.
• Optimal evaluation and management of patients with melanoma should include an assessment of individual social determinants of health.

## Introduction

The National Cancer Institute's Surveillance, Epidemiology, and End Results Program estimated 99,780 new cases of cutaneous melanoma in 2022, cementing its position as the fifth most prevalent cancer in the United States.^1^ Alarming projections indicate that by 2040, melanoma is poised to become the second most common malignancy and foremost cancer diagnosis among men.^2^ Equally concerning, melanoma accounts for the majority of reported skin cancer-related deaths, with an estimated 7990 melanoma-related fatalities in 2023.^3^ Historically, patients diagnosed with metastatic tumors faced a bleak prognosis, with a median survival of 9 months and a 5-year survival rate as low as 5%.^4^

While melanoma survival rates have improved in recent years, attributed in part to advances in research and treatment,^3^ disparities in survival persist among several patient subgroups.^5^, ^6^, ^7^ Historically, prognostic disparities have been identified along racial lines, with Black populations experiencing higher mortality rates compared to White populations and higher proportions of acral lentiginous melanoma.^7^^,^^8^ Paradoxically, non-Hispanic White patients exhibit a higher incidence of all skin cancers.^9^ Contributing to outcome disparities, growing evidence suggests race and ethnicity are associated with advanced stage at diagnosis and lower screening rates.^6^^,^^7^ The underlying causal factors driving these disparities remain understudied.

Social determinants of health (SDoH) are increasingly recognized as influential factors and circumstances affecting cancer treatment outcomes independent of tumor biology or therapeutic modalities. The World Health Organization defines SDoH as the conditions shaping daily life,^10^ and the US Department of Health and Human Services categorizes them into 5 major domains: health care access and quality, economic stability, social and community context, education access and quality, and neighborhood and built environment.^11^ Despite the pivotal role of SDoH in determining the burden of disease, little is known regarding the overall impact of each domain on melanoma treatment outcomes. Thus, this systematic review of observational manuscripts in the United States was conducted to evaluate the influence of SDoH on melanoma survival outcomes.

## Methods

The manuscript was registered with the Prospective Register of Systematic Reviews (CRD42022346854) and follows the Preferred Reporting Items for Systematic Reviews and Meta-Analyses guidlelines.^12^ On September 1, 2022, PubMed, Cochrane, EMBASE, Scopus, Web of Science, and Google Scholar were searched (Table I). Reference manager software (EndNote, Thomson Reuters) was used to remove duplicates. The references from identified manuscripts were manually screened for additional manuscripts.

**Table I:** Search strategies according to the database

Database	Search strategy
PubMed	("Cancer"[tiab]) **AND** ("Disparit∗"[tiab] **OR** ‴Social determin∗"[tiab] **OR** "Socio∗economic∗"[tiab] **OR** "Social Determinants of Health"[Mesh]) **AND** ("Survival Analysis"[Mesh] **OR** "Surviv∗"[Title/Abstract] **OR** "Recurre∗"[Title/Abstract] **OR** "Relapse"[Title/Abstract] **OR** "Prognos∗"[Title/Abstract] OR “Mortalit∗” **OR** “Outcome∗”[Title/Abstract]).
Cochrane	("Cancer"[ti,ab,kw]) **AND** ("Disparit∗"[ti,ab,kw] **OR** ‴Social determin∗"[ti,ab,kw] **OR** "Socio∗economic∗"[ti,ab,kw] **OR** "Social Determinants of Health"[Mesh]) **AND** "Surviv∗" **OR** "Recurre∗" **OR** "Relapse"**OR** "Prognos∗" **OR** “Mortalit∗” **OR** “Outcome∗”).
EMBASE	(“cancer”:ab,ti) **AND** (“disparit∗”:ab,ti **OR** “social determin∗”:ab,ti **OR** “socio∗economic∗”:ab,ti **OR** “social determinants of health”:ab,ti) **AND** (“Survival Analysis”:ab,ti **OR** “Surviv∗”:ab,ti **OR** “Recurre∗”:ab,ti **OR** “Relapse”:ab,ti **OR** “Prognos∗”:ab,ti **OR** “Mortalit∗” **OR** “Outcome∗”:ab,ti) **AND** [embase]/lim **AND** ([article]/lim **OR** [article in press]/lim).
SCOPUS	TITLE-ABS("cancer") **AND** TITLE-ABS("disparit∗" **OR** "Social determinan∗" **OR** "Socio∗economic∗" **OR** "Social Determinants of Health") **AND** TITLE-ABS("Survival Analysis" **OR** "Surviv∗" **OR** "Recurre∗" **OR** "Prognos∗" **OR** "Mortalit∗" **OR** "outcome∗") **AND** (LIMIT-TO [DOCTYPE,"ar"]) **AND** (LIMIT-TO [LANGUAGE,"English"])
Web of Science	(TI=[Cancer] **OR** AB=[Cancer]) **AND** (TI=["disparit∗" **OR** "Social determinan∗" **OR** "Socio∗economic∗" **OR** "Social Determinants of Health"] **OR** AB=["disparit∗" **OR** "Social determinan∗" **OR** "Socio∗economic∗" **OR** "Social Determinants of Health"]) **AND** (TI=["Survival Analysis" **OR** "Surviv∗" **OR** "Recurre∗" **OR** "Prognos∗" **OR** "Mortalit∗" **OR** "outcome∗"] **OR** AB=["Survival Analysis" **OR** "Surviv∗" **OR** "Recurre∗" **OR** "Prognos∗" **OR** "Mortalit∗" **OR** "outcome∗"])
Google Scholar	In the title of the article: Cancer **AND** "Social Determinants"

All searches were performed on September 1, 2022.

Inclusion criteria were based on Population, Exposure, Comparison, Outcome, and Manuscript Design. Manuscripts were excluded based on the following criteria: (1) outcomes not related to cancer survival, (2) pediatric patients, (3) results that could not be individualized (eg, only percentages presented), (4) patient data before January 2001 and after September 2022, (5) no actionable SDoH (eg, race and geography), (6) not evaluating melanoma, (7) case reports or literature reviews, (8) duplicates, and (9) patients from outside the United States (Table II).

**Table II:** Articles excluded and the reasons for exclusion (*n* = 24)

Reasons for exclusion	Author, year
1	13, 14, 15, 16, 17, 18, 19
2	^20^
3	21, 22, 23, 24, 25
4	26, 27, 28
5	^29^^,^^30^
6	^31^
7	^32^^,^^33^
8	^24^^,^^45^
9	^34^

1. Manuscripts that analyzed outcomes not related to cancer survival (*n* = 7).

2. Manuscripts that evaluated pediatric patients (*n* = 1).

3. Manuscripts for which results could not be individualized (*n* = 5).

4. Manuscripts using patient data before 2002 (*n* = 3).

5. Manuscripts without any actionable Social Determinants of Health (*n* = 2).

6. Manuscripts that did not evaluate melanoma (*n* = 1).

7. Case reports or literature reviews (*n* = 2).

8. Duplicates (*n* = 2).

9. Manuscripts evaluating patients outside the USA (*n* = 1).

In Phase I of selection, 2 authors (M.M.G. and K.T.) independently reviewed titles and abstracts and selected manuscripts that met the inclusion criteria. In Phase II, 2 authors (M.E.M. and C.B.) fully evaluated selected manuscripts by independently applying the inclusion and exclusion criteria. A third author and expert in clinical oncology (J.E.C.) was consulted if any disagreements arose in either phase. The following information was collected: manuscript characteristics (eg, author, year, country, and manuscript design); patient population characteristics (eg, sample size, participant age, and tumor stage); evaluated SDoH; survival outcomes; main results; and conclusions.

**Fig 1:**
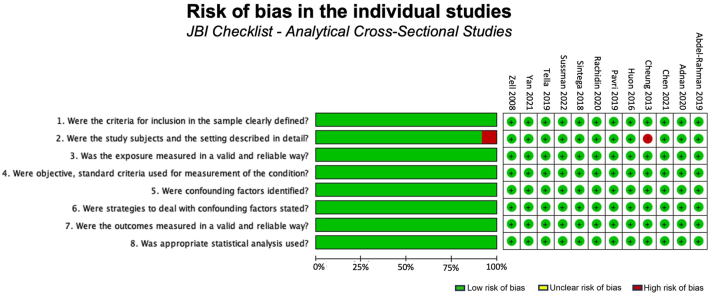
Risk of bias assessed by the Critical Appraisal Checklist for Analytical Cross-Sectional Manuscripts: author’s judgments for each included manuscript. “High” risk was denoted if 49% or less of the items were scored as yes, “moderate” if 50% to 69%, and “low” if 70% or more.

The risk of bias was assessed using the Critical Appraisal Checklist for Analytical Cross-Sectional Manuscripts^35^ by 2 authors (M.E.M. and C.B.) (Fig 1). A third author (K.T.) resolved any disagreements.

## Results

### Manuscript selection and characteristics

**Fig 2:**
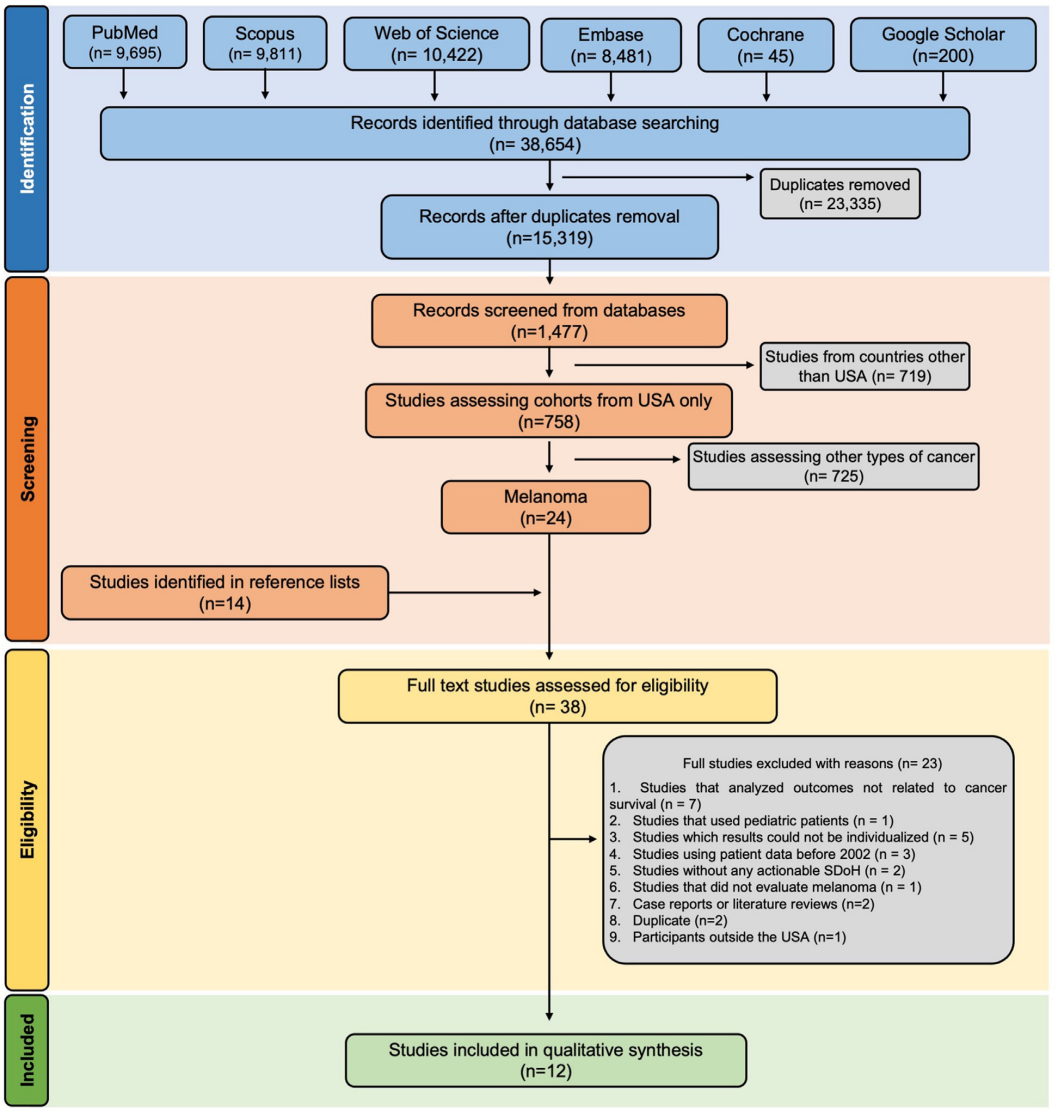
Flow diagram describing literature search and selection criteria adapted from the Preferred Reporting Items for Systematic Reviews and Meta-Analyses (PRISMA) guideline (2020).

**Fig 3:**
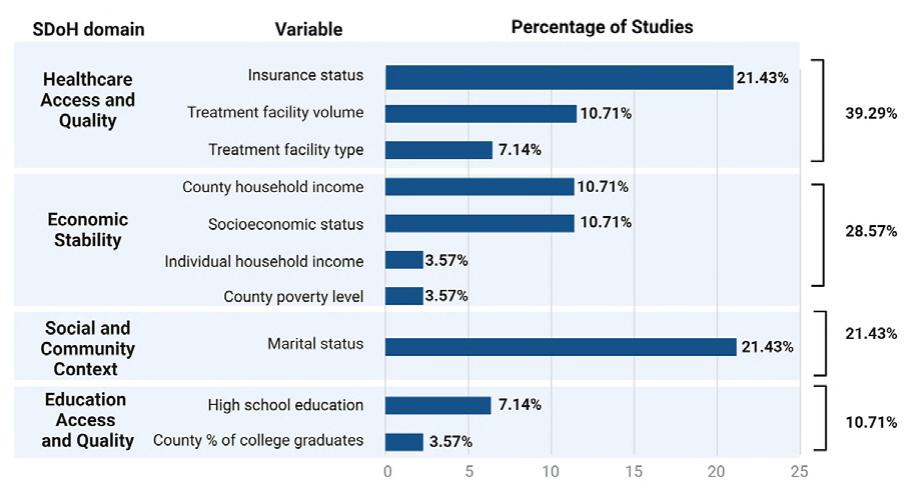
Distribution of the manuscripts according to the social determinant of health (SDoH) evaluated within the major domains.

An initial 15,319 records were identified; following the selection process, 12 manuscripts were included (Fig 2). Characteristics of the included manuscripts are summarized in Table III. This review encompassed 721,455 melanoma patients (ranging from 753 to 261,076 patients per manuscript). Most (*n* = 10) used data from national databases such as Surveillance, Epidemiology, and End Results Program (*n* = 8) and the National Cancer Database (*n* = 3). One manuscript utilized state-level data from the California Cancer Registry (*n* = 1); one utilized institutional data.^36^ The SDoH assessed by each manuscript is shown in Fig 3.

**Table III:** Main characteristics of included results

Variable	*N* (%) *n* = 12
Sample size	
Range	753-261,076
Mean (SD)	60,121 (70,904)
Median (IQR)	43,213 (71,998)
Manuscript setting	
National	10 (83.34)
State	1 (8.33)
Institutional	1 (8.33)
Data source	
SEER	8 (66.67)
NCDB	3 (25)
CCR	1 (8.33)
Melanoma clinical stage	
Stages I, II, III, and IV	6 (50)
Stages I, II, and III	1 (8.33)
Stages III and IV	2 (16.67)
Stage IV	2 (16.37)
Publication year	
2008-2012	1 (8.33)
2013-2017	2 (16.67)
2018-2022	9 (75)
Publication impact factor	
Range	2.3-50.7
Mean (SD)	11.49 (13.7)
Median (IQR)	7.25 (11.26)

*CCR*, California Cancer Registry; *IQR*, interquartile range; *NCDB*, National Cancer Database; *SEER*, Surveillance, Epidemiology, and End Results Program.

Regarding disease characteristics, half of the manuscripts included melanoma patients across all tumor stages, while one manuscript excluded stage IV patients.^37^ Two manuscripts specifically focused on advanced stages (III and IV),^36^^,^^38^ and 2 manuscripts restricted their sample to stage IV patients.^39^^,^^40^ The majority of the included manuscripts (*n* = 9) were published within a 4 year period (2018-2022). The journal impact factor of these publications exhibited a wide range, 2.3 to 50.7, with a mean value of 11.49 and a standard deviation of 13.7.

### Health care access and quality

Health care access and quality were evaluated through insurance status (*n* = 5, 50%)^38^^,^^39^^,^^41^, ^42^, ^43^ and treatment facility volume (*n* = 2, 16.7%)^38^^,^^40^ and type (*n* = 2, 16.7%).^39^^,^^41^ Two manuscripts (16.7%) analyzed melanoma-specific survival (MSS),^42^^,^^43^ and 4 (33.3%) evaluated overall survival (OS).^38^, ^39^, ^40^, ^41^

Insurance status was divided into no insurance, private insurance, and government-provided insurance. Four manuscripts identified a significant reduction in survival among individuals with government insurance or no insurance compared to those with private insurance.^38^^,^^39^^,^^41^^,^^42^ Conversely, only one manuscript found no significant difference in survival based on insurance status.^43^

**Fig 4:**
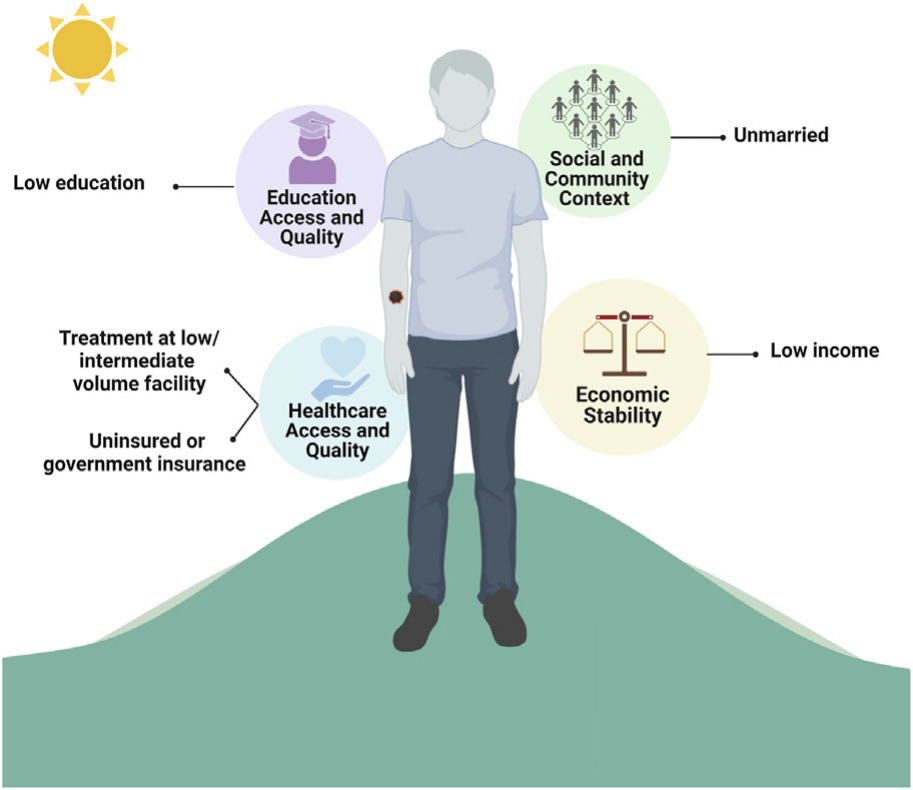
Social determinants of health predictors of worse melanoma-treatment outcomes.

Patients who received treatment at facilities with low or intermediate volume were consistently linked to worse OS (*n* = 2). Concerning treatment facility type, one manuscript found no correlation with melanoma outcomes,^41^ while another reported significantly increased OS when treatment was received at academic or research centers^39^ (Table IV and Fig 4).

**Table IV:** Summary of characteristics of included manuscripts evaluating association between social determinants of health and melanoma outcomes (*n* = 12)

Author (year)	Sample size	Tumor stage	Database	SDoH indicator	Outcome measure	Cohorts	HR (95% CI) or months	Prediction of worse outcome
Economic stability								
Yan et al (2022)^44^	2245	All∗	SEER	Socioeconomic status	MV MSS (HR)	Highest	Reference	Low SES
						High	1.08 (0.795-1.46)	
						Middle	1.09 (0.801-1.49)	
						Second-lowest	1.42 (1.028-1.96)†	
						Lowest	1.33 (0.903-1.96)	
Abdel-Rahman (2020)^45^	261,076	All‡	SEER	Socioeconomic STATUS	MV MSS (HR)	High	Reference	Low SES
						Middle	1.214 (1.178-1.251)†	
						Low	1.361 (1.315-1.409)†	
Zell (2008)^42^	39,049	All∗	CCR	Socioeconomic status	MV MSS (HR)	Lowest	Reference	Low SES
						Second-lowest	0.91 (0.79-1.06)	
						Middle	0.85 (0.74-0.98)†	
						High	0.72 (0.63-0.84)†	
						Highest	0.68 (0.59-0.78)†	
					MV OS (HR)	Lowest	Reference	Low SES
						Second-lowest	0.92 (0.83-1.00)	
						Middle	0.83 (0.76-0.91)†	
						High	0.76 (0.70-0.83)†	
						Highest	0.63 (0.58-0.69)†	
Sitenga and Aird (2018)^46^	80,907	All∗	NCBD	Household income by zip code	MV OS (HR)	>$63,000	Reference	Low household income
						$48,000-$62,999	1.11 (1.03-1.21)†	
						$38,000-$47,999	1.17 (1.07-1.28)†	
						<$38,000	1.24 (1.10-1.39)†	
Tella (2019)^38^	40,676	III∗	NCBD	Household income by zip code	MV OS	>$63,000	Reference	Low household income
						$48,000-$62,999	1.08 (1.02-1.14)†	
						$38,000-$47,999	1.11 (1.04-1.18)†	
						<$38,000	1.26 (1.17-1.36)†	
		IV∗				>$63,000	Reference	Low household income
						$48,000-$62,999	1.09 (1.02-1.16)†	
						$38,000-$47,999	1.09 (1.04-1.15)†	
						<$38,000	1.13 (1.08-1.19)†	
Adnan (2020)^43^	45,750	All‡	SEER	County household income	MV MSS (HR)	>58,000	Reference	No significant risk factor
						40,000-57,999	1.058 (0.928-1.207)	
						<40,000	1.210 (0.902-1.624)	
Cheung (2013)^41^	49,666	All‡	SEER	County household income	MV MSS (HR)	<$50k/year	Reference	Low household income
						≥$50k/year	0.115†	
Adnan (2020)^43^	45,750	All‡	SEER	County poverty % below federal poverty line	MV MSS (HR)	<10%	Reference	Increased poverty
						10-12.9%	0.998 (0.904-1.101)†	
						13-16.49% > 16.5%	1.113 (0.999-1.239)†	
							1.133 (0.973-1.319)†	
Health care access and quality								
Yan et al (2022)^44^	2245	All∗	SEER	Insurance status	MSS (HR)	Private or medicare	Reference	No significant risk factor
						Uninsured	0.46 (0.148-1.41)	
						Any Medicaid	0.85 (0.580-1.23)	
						Insured NOS	1.02 (0.683-1.54)	
Sussman (2022)^39^	24,544	IV∗	NCBD	Insurance status	MV OS (median months)	Government	7.62 (7.36-7.92)†	Government insurance and uninsured
						Not Insured	5.95 (5.36-6.67)†	
						Private	12.48 (12-12.98)†	
Adnan (2020)^43^	45,750	All‡	SEER	Insurance status	MV MSS (HR)	Insured	Reference	Government insurance and uninsured
						Medicaid	2.117 (1.928-2.324)†	
						Uninsured	1.938 (1.732-2.167)†	
Tella (2019)^38^	27,528	III∗	NCBD	Insurance status	MV OS	Private	Reference	Government insurance and uninsured
						Uninsured	1.48 (1.36-1.62)†	
						Medicaid	1.84 (1.69-2.01)†	
						Medicare	1.24 (1.17-1.31)†	
Tella (2019)^38^	13,148	IV∗	NCBD	Insurance status	MV OS	Private	Reference	Government insurance and uninsured
						Uninsured	1.44 (1.32-1.56)†	
						Medicaid	1.46 (1.34-1.57)†	
						Medicare	1.16 (1.10-1.22)†	
Sitenga & Aird (2018)^46^	80,907	All∗	NCBD	Insurance status	MV OS (HR)	Medicaid	Reference	Medicaid
						Uninsured	0.80 (0.68-0.93)†	
						Private	0.48 (0.42-0.54)†	
Sussman (2022)^39^	24,544	IV∗	NCBD	Treatment facility type	MV OS (median months)	Academic/research center	11.47 (11.1-11.99)†	Non-academic center
						Non-academic	7.89 (7.62-8.11)†	
Sitenga and Aird (2018)^46^	80,907	All∗	SEER	Treatment facility type	MV OS (HR)	Other cancer program	Reference	No significant risk factor
						Community cancer	1.03 (0.87-1.21)	
						Comprehensive cancer	1.02 (0.90-1.16)	
						Academic/research	0.94 (0.83-1.07)	
Tella (2019)^38^	27,528	III∗	NCBD	Treatment facility volume	MV OS (HR)	High	Reference	Low treatment facility volume
						Intermediate	1.15 (1.09-1.20)†	
						Low	1.23 (1.17-1.29)†	
Tella (2019)^38^	13,148	IV∗	NCBD	Treatment facility volume	MV OS (HR)	High	Reference	Intermediate treatment facility volume
						Intermediate	1.29 (1.23-1.36)†	
						Low	1.16 (1.10-1.21)†	
Huo et al (2016)^40^	1438	IV‡	SEER	Treatment facility volume	5-year OS (HR)	<5 patients	Reference	Low treatment facility volume
						6-10 patients	0.91 (0.80-1.02)	
						>10 patients	0.79 (0.69-0.90)†	
Education access and quality								
Adnan (2020)^43^	45,750	All‡	SEER	Percent of 25+ without high school diploma	MV MSS (HR)	<10%	Reference	No significant risk factor
						10-14.9%	0.966 (0.8780-1.061)	
						15-24.9%	1.073 (0.967-1.190)	
						>25%	1.131 (0.957-1.336)	
Sitenga and Aird (2018)^46^	80,907	All∗	NCBD	Proportion of a zip code without a high school diploma	MV OS (HR)	<7%	Reference	Increased proportion without high school diploma
						7-12.9%	1.12 (1.05-1.20)†	
						13-20.9%	1.21 (1.10-1.33)†	
						≥21%	1.21 (1.07-1.37)†	
Cheung (2013)^41^	49,666	All‡	SEER	County level % college graduate	MV MSS (HR)	≤25%	Reference	Low college graduation level
						>25%	0.114†	
Social and Community Context								
Yan et al (2022)^44^	2245	All∗	SEER	Marital status	MSS (HR)	Married	Reference	No significant risk factor
						Unmarried	1.26 (0.980-1.62)	
Chen et al (2021)^47^	98,801	All∗	SEER	Marital status	5-year MSS	Unmarried Male	Reference	Unmarried
						Married Male	1.42 (1.34-1.50)†	
						Unmarried Female	Reference	
						Married Female	1.35 (1.24-1.47)†	
Adnan (2020)^43^	45,750	All‡	SEER	Marital status	MV MSS (HR)	Unmarried	Reference	Unmarried
						Married	0.781 (0.732-0.833)†	
Rachidi et al (2020)^37^	73,558	III-IV§	SEER	Marital status	MV MSS (HR)	Married	Reference	Unmarried
						Single	1.49 (1.39-1.6)†	
						Divorced	1.37 (1.26-1.5)†	
						Widowed	1.45 (1.32-1.58)†	
Rachidi et al (2020)^37^	2992	III-IV§	John’s Hopkins	Marital status	MV OS (HR)	Married	Reference	Single and widowed
						Single	1.51 (1.15-1.99)†	
						Divorced	1.18 (0.72-1.92)	
						Widowed	1.54 (1.09-2.16)†	
Pavri et al (2019)^36^	753	I-III‖	SEER	Marital status	UV OS (HR)	Married	Reference	Widowed
						Single	0.62 (0.38-0.998)†	
						Widowed	1.60 (1.11-2.31)†	

*CCR*, California Cancer Registry; *HR*, hazard ratio; *MSS*, melanoma-specific survival; *MV*, multivariate; *NA*, data not available; *NCDB*, National Cancer Database; *OS*, overall survival; *SDoH*, social determinant of health; *SEER*, Surveillance, Epidemiology, and End Results Program; *SES*, socioeconomic status; *UV*, univariate.

∗ American Joint Committee on Cancer staging system.

† Authors considered significant.

‡ Stage based on SEER Registrar Staging Assistant.

§ Staging system not specified.

‖ Clark Level staging system.

### Economic stability

Economic stability was evaluated using county (*n* = 2, 16.7%)^42^^,^^45^ and zip code (*n* = 2, 16.7%)^38^^,^^41^ based household income, socioeconomic status (*n* = 3, 25%),^43^^,^^44^^,^^46^ and county poverty level (*n* = 1, 8.3%).^42^ MSS was evaluated in 5 manuscripts^42^, ^43^, ^45^, ^44^, ^46^; 3 evaluated OS.^38^^,^^41^^,^^46^ The majority of manuscripts (*n* = 7, 58.3%) found that low economic stability was significantly associated with reduced survival.

Of the 3 manuscripts that evaluated socioeconomic status, all had at least one group with decreased MSS compared to the highest group.^43^^,^^44^^,^^46^ Two of the manuscripts evaluated quintiles.^43^^,^^46^ In the first, compared to the lowest quintile, all but the second-lowest quintile had improved outcomes.^46^ In the other, compared to the highest quintile, the second-lowest group had significantly lower MSS.^43^ The third manuscript utilized tertiles and found that compared to the highest tertile, the other groups had significantly lower MSS.^44^

Increased household income determined by zip code improved survival (*n* = 2).^38^^,^^41^ When deriving household income from county-level data (*n* = 2), there were conflicting results. One manuscript found a significant decrease in MSS among lower county household incomes^45^ while the other demonstrated no significant difference in MSS^42^ (Table IV and Fig 4).

### Social and community context

Social and community context were exclusively assessed through marital status in 5 manuscripts (41.7%).^36^^,^^37^^,^^42^^,^^43^^,^^47^ All of these manuscripts included at least 2 marital status groups, married and unmarried; one manuscript also incorporated divorced and 2 widowed groups.^36^ Four manuscripts evaluated MSS (33.3%),^36^^,^^42^^,^^43^^,^^47^ and 2 measured OS (16.7%).^36^^,^^37^

Among these, the majority (*n* = 4) reported that compared to married individuals, being unmarried was significantly linked to decreased survival probability.^36^^,^^37^^,^^42^^,^^47^ Only one manuscript found no significant difference in MSS between married and unmarried groups.^43^ The one manuscript that included a divorced group reported mixed results. It found a significantly lower MSS with national data but no significant difference in OS with institutional data.^36^ Additionally, 2 manuscripts found that being widowed was significantly associated with lower survival compared to being married^37^^,^^38^ (Table IV and Fig 4).

### Education access and quality

Education access and quality were assessed by the population percentage without a high school diploma (*n* = 2, 16.7%)^41^^,^^42^ and the county percentage of college graduates (*n* = 1, 8.3%).^45^ Two manuscripts measured MSS,^42^^,^^45^ and one measured OS.^41^ While one manuscript found no significant correlation between a high school degree and melanoma outcomes,^42^ the other observed that individuals without a high school diploma had significantly decreased OS.^41^ Low college graduation rates were identified as a significant contributing factor for MSS in one manuscript^45^ (Table IV and Fig 4).

## Discussion

SDoH constitute a complex matrix of nonbiological variables and are potentially responsible for inequities in survival^48^; unfortunately, they are often overlooked in patient care. This systemic review identified meaningful relationships within various SDoH domains. Specifically, worse survival outcomes were correlated with lack of private insurance, treatment at low or intermediate-volume facilities, lower incomes, being unmarried, and lower education levels (Fig 4). These results represent opportunities for clinicians to incorporate SDoH into clinical practice and develop appropriate interventions to improve patient survival.

Regarding health care access and quality, this review found that compared to private insurance, other forms of insurance and being uninsured were related to worse melanoma survival outcomes. Previous studies have shown that patients with government insurance experienced delays in surgical resection of melanoma.^49^^,^^50^ Such delays in care increase the risk for disease progression. Further, patients with government insurance were less likely to receive immunotherapy, with a resultant increase in melanoma morbidity and mortality.^51^ Government insurance may have a more complex process for approving or granting access to certain therapies, delaying or compromising optimal care. These issues should be further examined and corrected.

In contrast, patients treated at higher-volume facilities and academic centers had better medical outcomes. Physicians at high-volume facilities are likely to have more experience resecting tumors and determining the most suitable treatment regimens.^52^ Further, high-volume facilities tend to follow evidence-based guidelines more frequently.^53^ Moreover, in-house access to more subspecialists may provide more cohesive multidisciplinary care, specifically improving outcomes through joint scientific discussions regarding patient-specific care.^54^ Lastly, higher-volume facilities are more likely to have increased access to clinical trials and cutting-edge therapies.^7^

Decreased levels of economic stability were associated with reduced melanoma survival. Many newer therapies, such as immunomodulators, decrease melanoma morbidity and mortality.^55^ However, these therapies are expensive, and low levels of economic stability are associated with lower levels of their use.^43^ For example, PD-1 inhibitors, a type of immunotherapy, can range in cost from $110,340 to $322,914.^56^ Further, these drugs are often used in tandem with other chemotherapeutics and medications. Thus, the combined cost of all treatments can be overwhelming. The monetary burden of these cancer medications has been coined “financial toxicity.”^57^ The financial implications of receiving these treatments include travel to specialized centers, housing away from home, lost income from jobs where sick leave is not offered, etc. Patients who initially may afford these treatment regimens are at risk of plunging into financial distress from the additive expenses over time. Financial toxicity has been associated with reduced quality of life and increased financial distress.^58^

Being married was a protective factor. A majority of melanomas are initially self-identified or identified by family members with self-screening often prompting evaluation.^59^ Women are particularly adept at self-screening for melanoma.^59^ Some anatomic sites, such as the back, are difficult to self-inspect and are more easily monitored with the help of a partner. However, the impact of marriage on melanoma survival must be explained by reasons other than just visual recognition by a spouse, since marital status was shown to have a significant impact across all anatomical sites, including areas easy to visualize.^36^ The mechanism by which marital status alters cancer outcomes might be the associated behavioral differences between married and single patients. Spouses may strongly encourage and schedule medical appointments for their partners in a timelier fashion. A spouse may strongly encourage protective measures against melanoma, such as the use of sunscreen.^60^ There is also the physical and emotional support offered by a companion during the course of complex and toxic therapies. Of note, multiple manuscripts suggest that marriage has a more robust protective effect from melanoma in men.^36^^,^^47^^,^^61^, ^62^, ^63^ Therefore, unmarried men would likely benefit from more frequent screenings to increase the odds of earlier detection. In general, partners advocate for the well-being of their loved ones, which may help explain the improvement in melanoma outcomes observed among married patients.^64^

Education access and quality were associated with melanoma survival outcomes, although there was some conflicting evidence. Overall, living in counties with low education levels was associated with lower melanoma survival rates. Surprisingly, the literature suggests that higher levels of education are associated with increased risk of developing melanoma.^65^, ^66^, ^67^ Individuals with a higher level of education tend to use sunscreens with a higher sun protection factor, but they also accrue more UV exposure than people with less education.^68^ While higher education was associated with increased melanoma incidence, lower levels of education were associated with increased morbidity and mortality from melanoma.^69^ A fundamental limitation of the evaluated manuscripts was the assessment of education through population-level variables, such as by county and zip code. Since these measures are not patient-specific, drawing definitive conclusions regarding patient educational level is challenging. The assessment of educational attainment may have served more as a proxy for economic stability, since higher levels of economic stability correlate with higher levels of education.^70^ Furthermore, although none of the included manuscripts assessed measures for education quality, it has been well established that people living in lower-income areas attend schools with fewer resources.^71^ Achieving a high school diploma from a school in a lower-income neighborhood might not yield the same literacy level and specifically health literacy as a high school diploma from a school in a higher-income neighborhood.

This manuscript has some limitations that should be considered. First, several measures evaluated in the included manuscripts were at a macro-level. Since not all the SDoH variables were patient-specific but the outcomes were patient-specific, confounding factors and generalizations will inevitably influence detected relationships. Second, SDoH are vast categories and only some variables were assessed. For example, social and community context was only evaluated as marital status. Although marriage is an important piece of social support, it cannot fully encompass a patient’s social support network. Further, no manuscripts were identified assessing neighborhood and built environment, a potentially important SDoH domain in the context of melanoma survival. Lastly, given the limited sample size evaluating each SDoH variable, these results should be interpreted carefully. Despite these potential limitations, this systematic review strongly emphasizes the critical role of SDoH in influencing melanoma survival.

This analysis revealed significant correlations between various SDoH domains and reduced melanoma survival, including low economic stability, government health insurance, lack of insurance, low educational attainment, and unmarried marital status. These findings highlight the pressing need for health care providers to acknowledge the substantial impact of SDoH on melanoma outcomes, prompting the adoption of a more comprehensive approach to patient-centered care. It is also important to assess and document SDoH during the evaluation of individual patients, and for manuscripts to include more, most or all SDoH to better understand the correlations among them. Integrating support mechanisms into clinical practice to bridge the gaps created by SDoH may promote equitable and effective interventions aimed at enhancing melanoma-related outcomes.

## Conflicts of interest

None disclosed.
